# Research trends and hotspots of glial fibrillary acidic protein within the area of Alzheimer’s disease: a bibliometric analysis

**DOI:** 10.3389/fnagi.2023.1196272

**Published:** 2023-09-27

**Authors:** Yutong Zou, Lei Li, Lihua Guan, Chaochao Ma, Songlin Yu, Xiaoli Ma, Chenhui Mao, Jing Gao, Ling Qiu

**Affiliations:** ^1^Department of Laboratory Medicine, Peking Union Medical College Hospital, Peking Union Medical College and Chinese Academy of Medical Science, Beijing, China; ^2^Medical Science Research Center (MRC), Peking Union Medical College Hospital, Peking Union Medical College and Chinese Academy of Medical Sciences, Beijing, China; ^3^Department of Neurology, Peking Union Medical College Hospital, Peking Union Medical College and Chinese Academy of Medical Sciences, Beijing, China; ^4^State Key Laboratory of Complex Severe and Rare Diseases, Peking Union Medical College Hospital, Peking Union Medical College and Chinese Academy of Medical Sciences, Beijing, China

**Keywords:** Alzheimer’s disease, glial fibrillary acidic protein, bibliometric approach, research trend analysis, body fluids

## Abstract

**Objective:**

Our aim was to analyze the trends and hotspots on glial fibrillary acidic protein (GFAP) within the area of Alzheimer’s disease (AD) by using a bibliometric method, which is currently missing.

**Methods:**

All articles and reviews on GFAP within the area of AD from inception to December 31, 2022, were searched from the Web of Science Core Collection. Full records were derived, imported into Microsoft Excel, and analyzed by BIBLIOMETRC, VOSviewer, and CiteSpace.

**Results:**

In total, 2,269 publications, including 2,166 articles, were ultimately included. The number of publications from 81 countries/regions and 527 academic journals increased annually. The top three prolific countries and institutions were the USA, China, and England, the University of Gothenburg (Sweden), Federal University of Rio Grande do Sul (Brazil), and UCL Queen Square Institute of Neurology (England). Henrik Zetterberg from the University of Gothenburg, Kaj Blennow from the University of Gothenburg, and Alexei Verkhratsky from the University of Manchester were the top three prolific and cited authors; Journal of Alzheimer’s Disease, Brain Research, and Neuroscience contributed the most publications. The top key areas of research included “molecular, biology, and genetics” and “molecular, biology, and immunology,” and the top published and linked meaningful keywords included oxidative stress, inflammation/neuroinflammation, microglia, hippocampus, amyloid, cognitive impairment, tau, and dysfunction.

**Conclusion:**

Based on the bibliometric analysis, the number of publications on GFAP within the area of AD has been rapidly increasing, especially in the past several years. Oxidative stress and inflammation are research hotspots, and GFAP in body fluids, especially blood, could be used for large-scale screening for AD.

## 1. Introduction

As the most common form of dementia, Alzheimer’s disease (AD) comprises 60–80% of all dementia cases. Worldwide, the numbers of patients with AD are expected to reach 65.7 million in 2030 and 115.4 million in 2050, with a rate of one new case every 3 s (Prince et al., 2013). As a fatal illness, AD’s average survival time ranges from 4 to 8 years for patients aged 65 years and older (Larson et al., 2004). Since AD is still not curable, early diagnosis and intervention are critical to slow the development of AD and improve the quality of life of patients. In addition to the classical biomarkers recommended by guidelines (Zou et al., 2023), many other biomarkers in cerebrospinal fluid (CSF) and/or serum/plasma, such as neurofilament light (NfL), glial fibrillary acidic protein (GFAP), and alpha-synuclein, were recently recommended for the early and differential diagnosis of AD (Teunissen et al., 2022; Johnson et al., 2023).

As an intermediate filament structural protein involved in cytoskeleton assembly and integrity, GFAP is highly expressed in activated glial cells (Ganne et al., 2022). Thus, GFAP serves as a biomarker of neurodegenerative diseases, including AD, dementia with Lewy bodies, and frontotemporal dementia. In recent years, an increasing number of studies have aimed to explore the effect of GFAP on the development, diagnosis, and prognosis of AD. GFAP in body fluids, including CSF and serum/plasma, is considered a good biomarker for the early diagnosis and prognosis of AD, especially blood GFAP, which is considered an excellent biomarker for large-scale screening (Heimfarth et al., 2022; Teunissen et al., 2022; Guo et al., 2023). Moreover, it was reported that GFAP plays important roles in AD development, such as neuropathic aggregate accrual, and could become a novel promising therapeutic target to alleviate, delay, and even prevent AD (Ganne et al., 2022). However, GFAP is not specific for AD but is related to many neurological diseases (Heimfarth et al., 2022). Thus, it is still urgent to further explore the relationship between GFAP and AD and clarify the effect of GFAP on the occurrence, development, diagnosis, and prognosis of AD.

Thus, we conducted the first bibliometric analysis to identify research trends and hotspots on GFAP within the area of AD to provide a comprehensive understanding of this topic and further guide future research directions.

## 2. Methods

### 2.1. Search strategy

All data were collected from the Science Citation Index Expanded (SCI-E) of the Web of Science Core Collection database (WoSCC). The search strategy was as follows: ((TS = (GFAP)) OR TS = (glial fibrillary acidic protein)) AND ((((((TS = (alzheimer)) OR TS = (alzheimer’s)) OR TS = (alzheimer-disease)) OR TS = (alzheimers)) OR TS = (alzheimers-disease)) OR TS = (AD)). Only articles and reviews from inception to December 31, 2022, were included in this study. No language and region were restricted.

### 2.2. Analytical tool

Full records and cited references of all publications were derived from the WoSCC database. Important bibliometric parameters such as title, publication year, author, institution, keywords, journal, and citation were extracted, imported into Microsoft Excel 16.0 (Redmond, WA, US), and analyzed by the BIBLIOMETRC Online analysis platform^1^, VOSviewer (version 1.6.18), and CiteSpace (version 6.1 R3) software. The data on publication trends, countries/regions, and annual hot keywords were analyzed by the BIBLIOMETRC Online analysis platform, interdisciplinary relationships, and the top 15 keywords were analyzed by CiteSpace software, and other analyses were conducted by VOSviewer software.

## 3. Results

### 3.1. General publication trends

**Figure 1:**
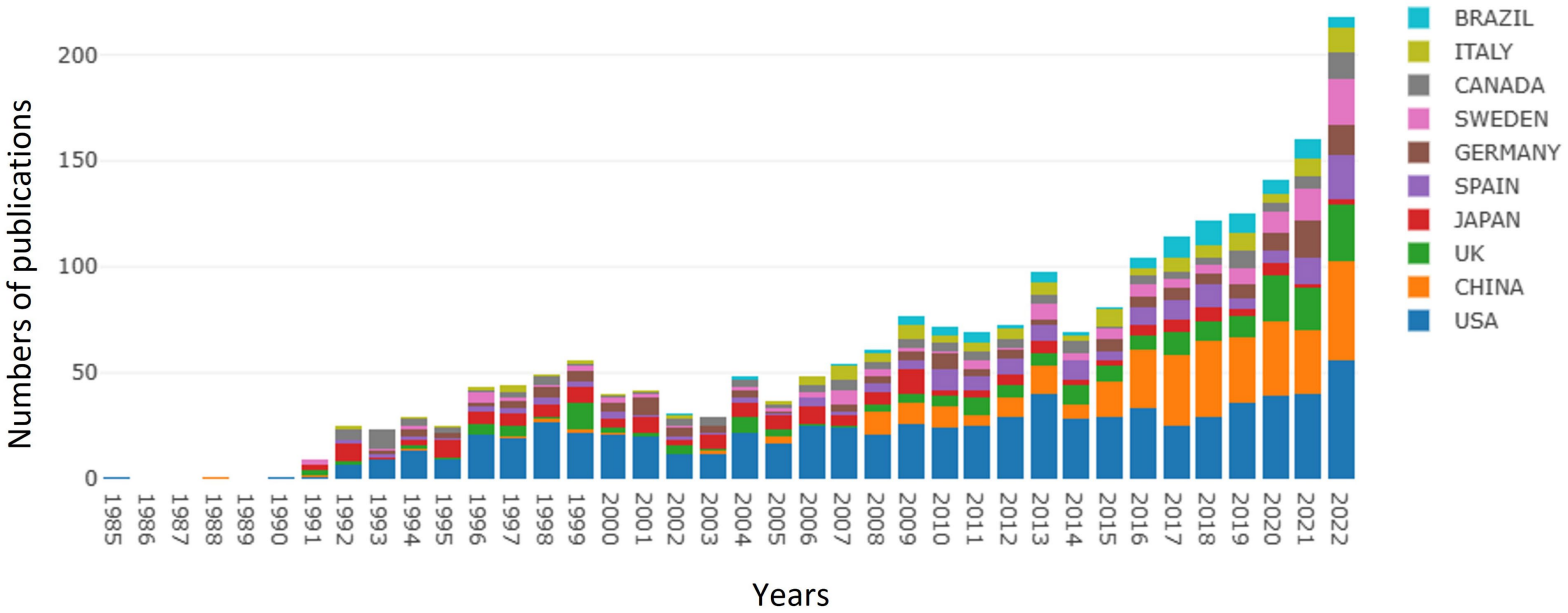
Summary of annual numbers of publications from different countries/regions.

As of December 31, 2022, a total of 2,269 publications about GFAP within the area of AD were recognized, of which 2,166 publications were recognized as articles, and others were reviews. As shown in Figure 1, an overall upward trend for the number of publications on GFAP within the area of AD was found despite some minor fluctuations. Since 2014, the upward trend has been successive and remarkable, which means that increasing attention has been given to GFAP within the area of AD in recent years. Moreover, studies focused on this area have also become a hotspot in China, with remarkably more publications in recent years.

### 3.2. Analysis of countries/regions

**Figure 2:**
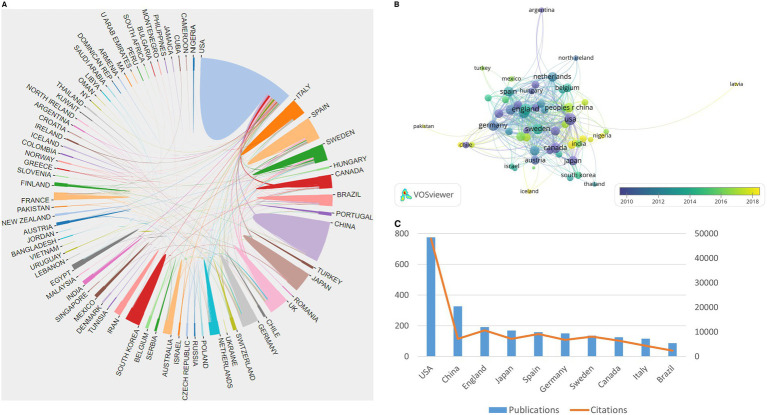
Summary of dominant countries/regions. **(A)** Collaboration among all countries; **(B)** Collaboration among 57 countries with a minimum number of documents of a country equal to three; the node size is based on the number of articles, and the total link strength is based on the power of cooperation between two nodes; **(C)** The publications and citations of the top 10 contributing countries.

Based on the VOSviewer software, the international collaboration among different countries/regions in this field is depicted in Figure 2A. With the minimum number of documents of a country set at three, 57 of 81 countries/regions were selected for visualization (Figure 2B). The USA, China, England, Japan, Spain, Germany, and Sweden are the largest nodes and are more broadly connected, indicating their close collaboration and significant academic influence in the field. Moreover, the top 10 contributing countries are shown in Figure 2C. The USA contributed the most publications in the field, with 776 publications and 47,971 total citations. China ranked second, with 326 publications and 7,181 total citations, and England ranked third, with 192 publications and 10,612 total citations.

### 3.3. Analysis of institutions

**Figure 3:**
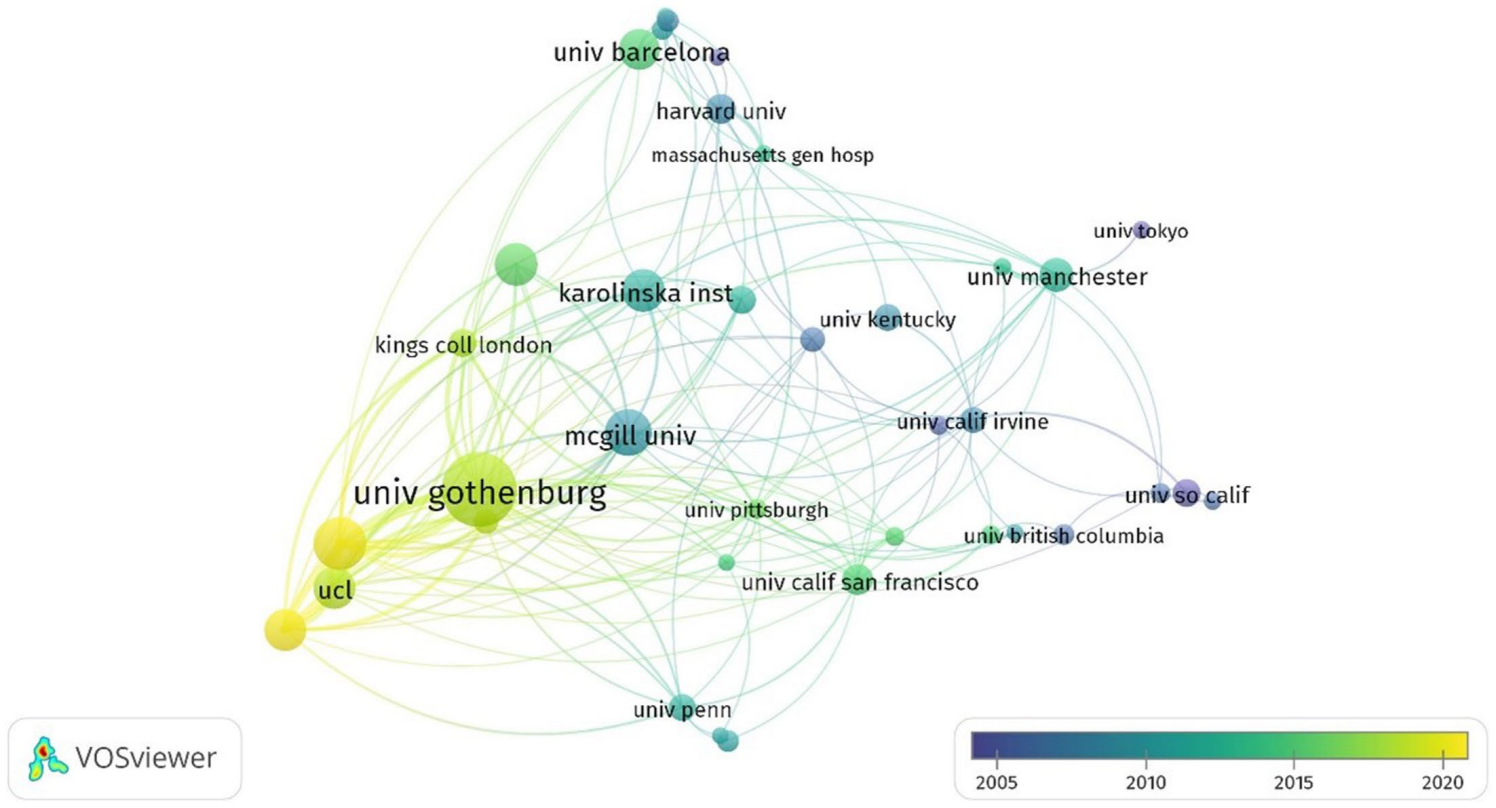
Summary of dominant institutions. The node size is based on the number of articles, and the total link strength is based on the power of cooperation between two nodes.

As shown in Table 1, the top 10 most prolific and highly cited institutions focused on GFAP within the area of AD are listed. The University of Gothenburg (Sweden) ranked first, with 91 publications and 475 total citations; the Federal University of Rio Grande do Sul (Brazil) ranked second, with 65 publications and 200 total citations; and the UCL Queen Square Institute of Neurology ranked third (England), with 63 articles. Of the top 10 institutions, three institutions are located in Sweden, and another three institutions are located in the USA. Figure 3 displays the network of collaborations among 41 dominant institutions with the minimum number of documents of an institution equal to 15. As one of the most central nodes, the University of Gothenburg (Sweden) contributed most publications, focused on research frontiers, and closely collaborated with many other institutions, including McGill University (Canada), the University of Barcelona (Spain), and the University of California, San Francisco (USA).

**Table 1:** The top 10 most prolific and highly cited institutions.

Institution	Country	Publications	Total citations	Average citations
Univ Gothenburg	Sweden	91	475	5.22
Federal University of Rio Grande do Sul	Brazil	65	200	3.08
McGill Univ	Canada	64	193	3.02
Univ Barcelona	Spain	64	108	1.69
Univ Calif San Francisco	USA	54	117	2.17
Univ Kentucky	USA	50	160	3.20
Sahlgrens Univ Hosp	Sweden	47	112	2.38
UCL	England	46	132	2.87
Karolinska Inst	Sweden	45	148	3.29
Univ Penn	USA	40	102	2.55

### 3.4. Analysis of authors

**Figure 4:**
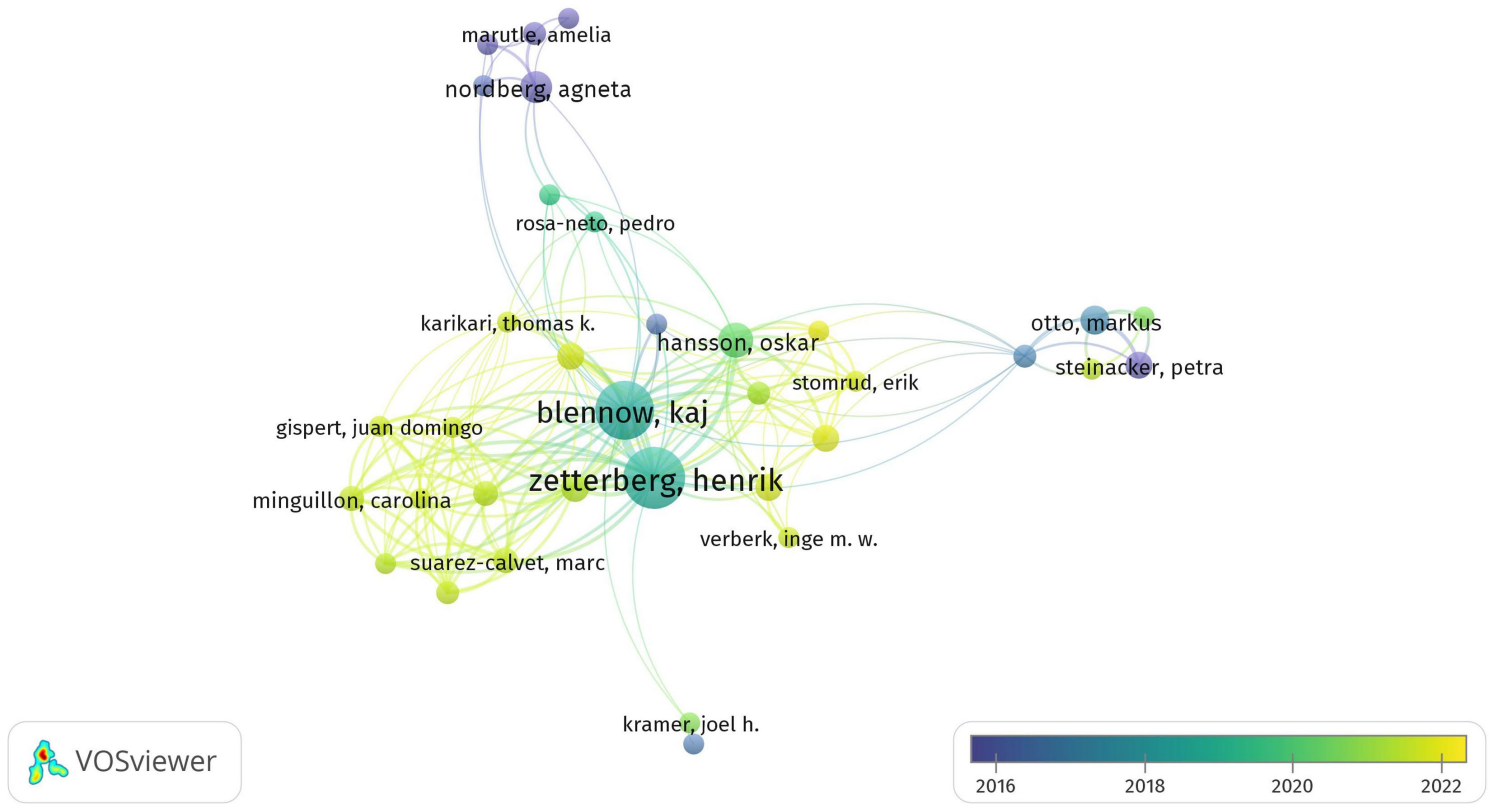
Summary of the dominant authors. The node size is based on the number of articles, and the total link strength is based on the power of cooperation between two nodes.

In total, 12,559 authors contributed to the publications on GFAP within the area of AD. The top 127 prolific and highly cited authors with at least five citations and their correlations are shown in Figure 4. The top three prolific and most cited authors were Henrik Zetterberg from the University of Gothenburg (publications, *n* = 52; total citations, *n* = 2,273); Kaj Blennow from the University of Gothenburg (publications, *n* = 45; total citations, *n* = 2,217); and Alexei Verkhratsky from the University of Manchester (publications, *n* = 17; total citations, *n* = 2,269). Of all the authors, Kaj Blennow and Henrik Zetterberg made more remarkable contributions in this area, with significantly higher total link strengths.

### 3.5. Analysis of journals and top 10 most cited publications

All the retrieved publications were from 527 different journals, and the top 10 most prolific journals are listed in Table 2. The top five journals in terms of number of publications were Journal of Alzheimer’s Disease (78 publications), Brain Research (69 publications), Neuroscience (63 publications), Neurobiology of Aging (58 publications), and Journal of Neuroinflammation (55 publications); the top five cited journals were Journal of Neuroscience (5,915 citations), Neurobiology of Aging (4,598 citations), Neuroscience (4,193 citations), Brain Research (3,680 citations), and Journal of Alzheimer’s Disease (3,059 citations). Of all 527 journals, the Journal of Alzheimer’s Disease contributed to the most total link strength for the publications on GFAP within the area of AD. Moreover, the top 10 most cited publications are shown in Table 3, seven of which are review publications. The most cited publication (Olsson et al., 2016) was a systematic review and meta-analysis, which summarized the diagnostic performance of common CSF and blood biomarkers for AD. However, published articles about the diagnostic performance of CSF and/or blood GFAP at that time were still limited; thus, new meta-analysis is still needed.

**Table 2:** The top 10 most prolific journals.

Journals	Publications	Total citations	Average citations
Journal of Alzheimer’s disease	78	231	2.96
Brain Research	69	201	2.91
Neuroscience	63	150	2.38
Neurobiology of Aging	58	394	6.79
Journal of Neuroinflammation	55	86	1.56
Experimental Neurology	49	132	2.69
Acta Neuropathologica	45	131	2.91
Neuroscience Letters	44	129	2.93
Journal of neuroscience research	41	119	2.90
Journal of Neurochemistry	38	109	2.87

**Table 3:** The top 10 most cited publications.

First author	Year	Journal	Country	Type	Citation
Bob Olsson (Olsson et al., 2016)	2016	Lancet Neurology	Sweden	review	1,038
Milos Pekny (Pekny et al., 2014)	2014	Neuroscience Letters	Australia	review	454
Elly M. Hol (Hol and Pekny, 2015)	2015	Current Opinion in Cell Biology	Netherlands	review	420
Vladimir Parpura (Parpura et al., 2012)	2012	Journal of Neurochemistry	USA	review	388
Pradip K. Kamat (Kamat et al., 2016)	2016	Molecular Neurobiology	USA	article	277
Md Sahab Uddin (Uddin et al., 2018)	2018	Frontiers in Aging Neuroscience	Bangladesh	review	207
Douglas M. Zeppenfeld (Zeppenfeld et al., 2017)	2017	JAMA Neurology	Portland	article	203
Kevin K. Wang (Wang et al., 2018)	2018	Expert Review of Molecular Diagnostics	USA	review	195
Tahir Muhammad (Muhammad et al., 2019)	2019	Nutrients	Korea	article	191
Georgia R. Frost (Frost and Li, 2017)	2017	Open Biology	USA	review	184

### 3.6. Analysis of research areas and keywords

**Figure 5:**
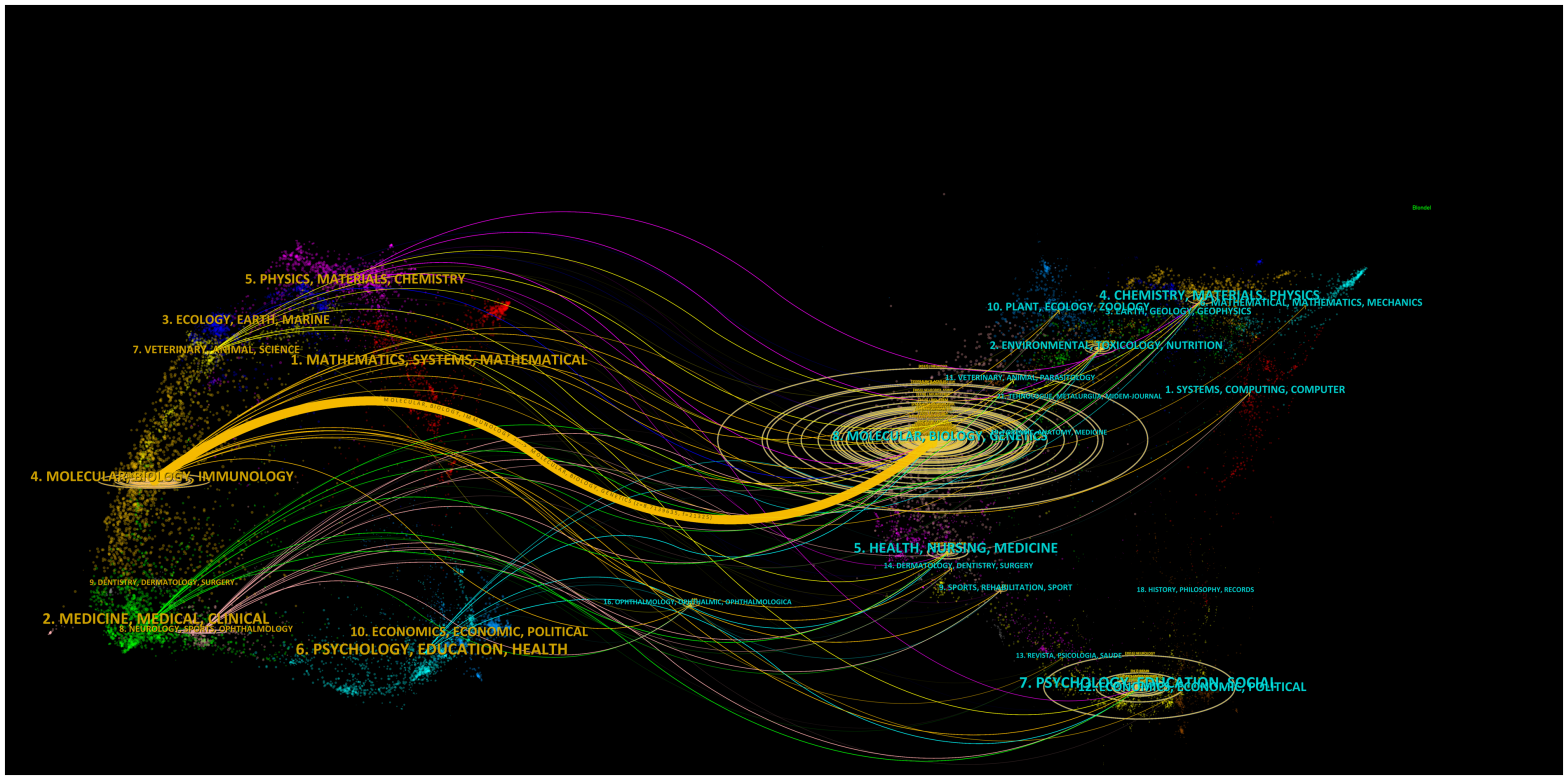
The dual-map overlay of the discipline distribution of publications. The left part represents citing journals, and the right part represents cited journals; the lines represent relationships between the citing and cited journals; the horizontal axis of the ellipse shows the number of authors, while the vertical axis shows the number of published articles.

As shown in Figure 5, the dual-map overlay of the discipline distribution of publications revealed the basis of discipline orientation and interaction between disciplines. Of 21 disciplines, “molecular, biology, genetics,” “molecular, biology, immunology”; and “psychology education social” were the key research areas. In addition, the published articles on “molecular, biology, immunology” were based on those on “molecular, biology, genetics,” suggesting that “genetics” had provided a foundation for this area, especially the “immunology” discipline.

**Figure 6:**
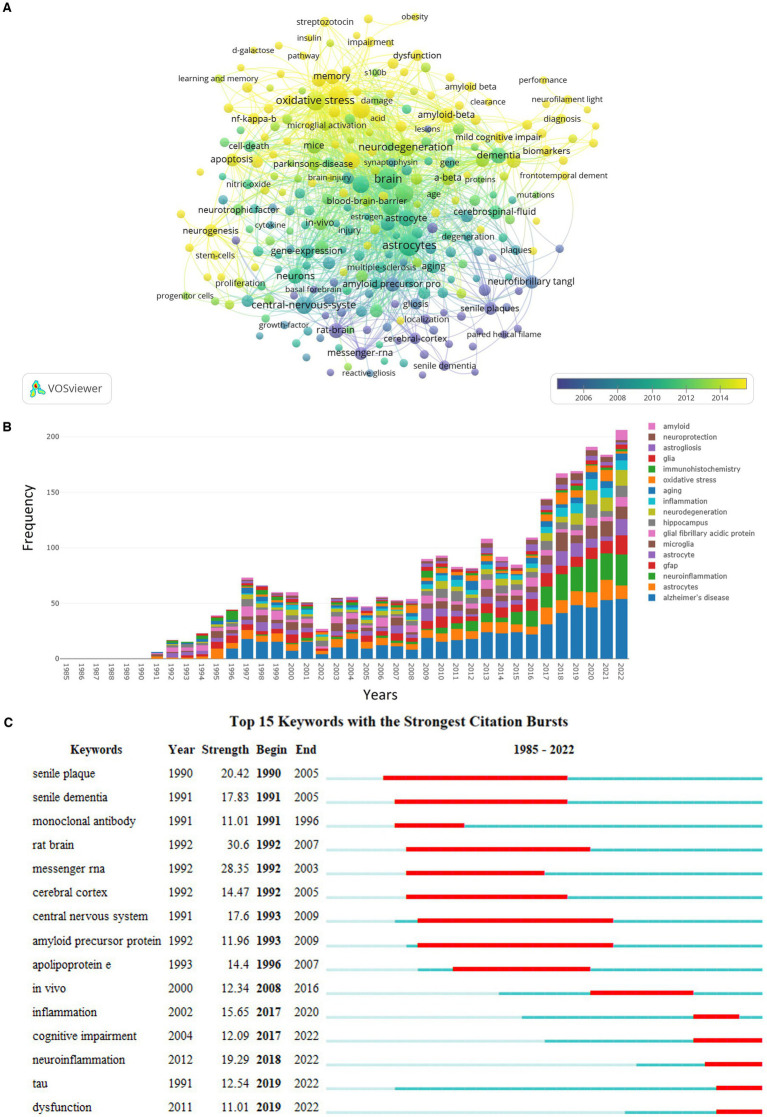
Summary of keywords. **(A)** Relationship among 285 keywords with a minimum number of occurrences equal to 15; the node size is based on the number of articles, and the total link strength is based on the power of cooperation between two nodes; **(B)** the annual frequency of top prolific keywords; **(C)** the top 15 keywords with the strongest citation bursts.

In total, 8,891 keywords were recognized from previous publications, 285 of which occurred at least 15 times. The co-occurrence of these 285 keywords is shown in Figure 6A. Of all keywords, the most published and linked meaningful keywords included “astrocytes,” “brain,” “oxidative stress,” “neuroinflammation,” “microglia,” “inflammation,” “neurodegeneration,” “central nervous system,” “hippocampus,” and “mouse model.” As shown in Figure 6B, the number of publications focused on the association between GFAP and “aging,” “oxidative stress,” “inflammation,” “microglia,” “hippocampus,” and “amyloid” within the area of AD annually increased in general, and “immunohistochemistry” was applied more for this area. Moreover, “inflammation,” “cognitive impairment,” “neuroinflammation,” “tau,” and “dysfunction” were the hot associated keywords in the past 5 years (Figure 6C).

## 4. Discussion

To analyze the research trends and hotspots on GFAP within the area of AD, we conducted a bibliometric analysis and eventually included 2,269 articles and review publications. To our knowledge, this is the first bibliometric analysis to explore the research on GFAP and AD. Despite some minor fluctuations, the general trend for the number of publications was upward, especially from 2014, when the upward trend became successive and remarkable. Around this time, the diagnostic performance of GFAP in CSF and blood was explored based on biochip array technology (Rosén et al., 2011). Then, the most cited publication (Olsson et al., 2016) further evaluated the diagnostic performance of many CSF and blood biomarkers for AD by conducting a meta-analysis, including GFAP in CSF, which implied the importance of GFAP for the diagnosis of AD. However, related articles about GFAP in CSF for the diagnosis of AD were still limited at that time, and only two articles were included in the meta-analysis; thus, a null conclusion was reported. Moreover, the application of GFAP in serum/plasma in AD had not been comprehensively recognized at that time, thus this review did not include it. During the past few years, the diagnostic value of GFAP in body fluids, especially in blood, was further explored and it could be used for large-scale screening (Guo et al., 2023). Although astrocyte activation is not specific to AD pathophysiology, plasma GFAP was found to be more sensitive for the early diagnosis of AD than Aβ-PET, CSF and plasma p-tau181, and CSF total tau (Guo et al., 2023), and it linearly increased with increasing Aβ-PET burden (Asken et al., 2020). Blood GFAP levels can predict not only AD development in patients with mild cognitive impairment but also cognitive decline in cognitively unimpaired subjects (Verberk et al., 2020; Cicognola et al., 2021; Verberk et al., 2021). At the same time, more sensitive technology, such as ultrasensitive single-molecule array technology, was also developed (Rissin et al., 2010) and applied for the accurate measurement of blood GFAP (Bogoslovsky et al., 2017), which prompted its application. Under these circumstances, the diagnostic and prognostic performance of GFAP in CSF and serum/plasma for AD should be further evaluated and verified with better analytical assays in larger-scale cohorts, especially for different age groups and clinical contexts (Hansson et al., 2022). Moreover, new systematic reviews and meta-analyses based on more publications, which are currently lacking, need to be conducted.

Interestingly, it is reported that the association of plasma GFAP with Aβ pathology appears stronger than that of CSF GFAP, which is different from other glial biomarkers (Pereira et al., 2021). A recent study found that plasma GFAP is more stable than CSF GFAP, without the effect of freeze–thaw cycles, making it a better biomarker for clinical application (Simrén et al., 2022). However, the reason why blood GFAP showed a stronger association with Aβ pathology is still unclear; thus, further work is still needed to investigate the mechanisms of GFAP release into the bloodstream under pathological conditions and clarify the key pathological substrates of the better separation of serum/plasma versus CSF in AD (Hansson et al., 2022). Moreover, even if the Alzheimer’s Association has recommended plasma GFAP as a promising and available biomarker since 2022 (Hansson et al., 2022; Teunissen et al., 2022), many problems still need to be solved, such as preanalytical confounders and biological factors. It was found that sample types with different anticoagulants could affect the concentration of blood GFAP, but centrifugation delay within 24 h, storage delay within 2 w at −20°C, centrifugation temperature and freeze–thaw cycle did not significantly affect plasma GFAP (Verberk et al., 2022). Additionally, significant differences were found for many AD-related biomarkers, including GFAP, between the postprandial group and fasting group, implying that the concentration of these biomarkers could be affected by food intake (Huber et al., 2023). Moreover, the concentration of blood GFAP is usually simultaneously measured with other biomarkers, such as amyloid and tau; thus, a standardized preanalytical process including sample drawing, treatment, and storage is still necessary (Verberk et al., 2022). Furthermore, it is important to evaluate the effects of clinical confounders and biological factors on the clinical diagnostic and prognostic performance of GFAP for AD (Hansson et al., 2022). Previous studies preliminarily found that impaired kidney function could affect the concentration of amyloid 1–42, p-tau181, and NfL but not plasma GFAP (Sedaghat et al., 2023; Zhang et al., 2023); however, more comprehensive studies with larger cohorts are still needed to explore the effect of more factors, such as peripheral neuropathies and body mass index, on blood GFAP for more accurate laboratory measurements and clinical interpretation (Hansson et al., 2022).

By this bibliometric analysis, it was also found that the USA, China, England, Japan, Spain, Germany, and Sweden contributed the most research on GFAP within the area of AD. The USA always took the lead in this area, but publications from China and England increased annually. As the top populous country with a rapidly increasing aging population, China has an increasing number of patients diagnosed with AD (Jia et al., 2020), and GFAP in CSF and serum/plasma has become a research hotspot, showing promising early diagnosis and prognosis value for AD. Of the top 10 prolific and highly cited institutions, three institutions are from the USA, and another three institutions are from Sweden, of which the University of Gothenburg contributed most publications in this area. Moreover, Henrik Zetterberg and Kaj Blennow, both professors at the University of Gothenburg, contributed the most prolific publications. As key leaders of the UK Dementia Research Institute and the Alzheimer’s Association, respectively, Henrik Zetterberg and Kaj Blennow focused on the fluid biomarkers of neurodegenerative diseases and made remarkable contributions in this area. Moreover, the Journal of Alzheimer’s Disease published most articles and reviews in this area, and the publications of Neurobiology of Aging had the highest average citations.

The discipline distribution analysis of publications revealed that “molecular, biology, genetics” and “molecular, biology, immunology” were the key research areas of 21 domain disciplines, and the published articles on “molecular, biology, immunology” were based on those on “molecular, biology, genetics,” which implied that “molecular, biology, genetics” had provided a foundation for this research area. Furthermore, the most published and hottest meaningful associated keywords included “oxidative stress,” “neuroinflammation” or “inflammation,” “microglia,” “neurodegeneration,” “hippocampus,” “mouse model,” “amyloid,” “immunohistochemistry,” of which “inflammation,” “cognitive impairment,” “neuroinflammation,” “tau,” and “dysfunction” were the hot associated keywords in the past 5 years. Because it is difficult to directly intervene in the human body and obtain human brain tissues, animal models especially mouse models are helpful and necessary for associated immunology and genetics studies (Qin et al., 2020), and immunohistochemistry remains the gold-standard technique for capturing the spatial expression patterns of astrocytes in postmortem tissue sections (Viejo et al., 2022). Since astrocytes and microglia could release proinflammatory cytokines and reactive oxygen, triggering neuroinflammation and oxidative stress (Novoa et al., 2022), it is not surprising that “inflammation,” “neuroinflammation,” and “oxidative stress” were hot keywords related to GFAP, which is an important biomarker for astrocyte activation. Moreover, the inflammatory response, oxidative stress, and glial activation (especially microglia and astrocytes) have been found as three main factors for AD progression based on both mouse models and human post-mortem brains (Companys-Alemany et al., 2022; Viejo et al., 2022). Furthermore, Aβ and tau are found able to activate astrocytes and microglia, and GFAP expression and protein concentrations were higher in areas surrounding dense Aβ plaques and increased with tau accumulation, implying the association of GFAP with amyloid and tau pathology (Simpson et al., 2010; Novoa et al., 2022). However, the results based on clinical individuals only showed a significant association between higher CSF and plasma GFAP levels and elevated Aβ-PET load but not for tau-PET load (Pereira et al., 2021; Ferrari-Souza et al., 2022). Moreover, it was found that glial biomarkers including GFAP increased with aging regardless of Aβ status and also in other neurological diseases besides AD (Milà-Alomà et al., 2020). Thus, the mechanisms of GFAP in the development of AD are complex due to the multiple functions of astrocytes, and more mechanism studies are still needed to clarify the specific causal relationship between GFAP and AD. Noticeably, all findings based on animal models need to be further verified based on human cohorts to ensure the clinical application.

In summary, the effects of GFAP on the occurrence, development, and prognosis of AD still need to be further researched, and our analysis could provide some guidance on further research trends. It should be emphasized that the GFAP change is not specific for AD but for many neurodegenerative diseases. Thus, GFAP in body fluid could only be used for the first screening but not a final diagnosis.

## Author contributions

YZ: methodology, data curation, and writing – original draft preparation. LL, LG, and CCM: methodology and data curation. SY, XM, CHM, and JG: writing – reviewing and editing. LQ: conceptualization and methodology. All authors contributed to the article and approved the submitted version.

## Footnotes

^1^ https://bibliometric.com/#home
