# Corrigendum: Research trends and hotspots of glial fibrillary acidic protein within the area of Alzheimer's disease: a bibliometric analysis

**DOI:** 10.3389/fnagi.2023.1312361

**Published:** 2023-10-31

**Authors:** Yutong Zou, Lei Li, Lihua Guan, Chaochao Ma, Songlin Yu, Xiaoli Ma, Chenhui Mao, Jing Gao, Ling Qiu

**Affiliations:** ^1^Department of Laboratory Medicine, Peking Union Medical College Hospital, Peking Union Medical College and Chinese Academy of Medical Science, Beijing, China; ^2^Medical Science Research Center (MRC), Peking Union Medical College Hospital, Peking Union Medical College and Chinese Academy of Medical Sciences, Beijing, China; ^3^Department of Neurology, Peking Union Medical College Hospital, Peking Union Medical College and Chinese Academy of Medical Sciences, Beijing, China; ^4^State Key Laboratory of Complex Severe and Rare Diseases, Peking Union Medical College Hospital, Peking Union Medical College and Chinese Academy of Medical Sciences, Beijing, China

**Keywords:** Alzheimer's disease, glial fibrillary acidic protein, bibliometric approach, research trend analysis, body fluids

In the published article, there was an error in ‘Table 1 The top 10 most prolific and highly cited institutions’, as published. It has been noted by a reader that the article mistakenly refers to Brazil (the country) as Brasilia (the capital city of Brazil). The corrected context of the Table 1 appears below.

“Federal University of Rio Grande do Sul (Brazil).”

In the published article, there was an error. It has been noted by a reader that the article mistakenly refers to Brazil (the country) as Brasilia (the capital city of Brazil).

A correction has been made to the abstract: Results and section ‘2. Methods’ and ‘3.3. Analysis of institutions’. This sentence previously stated:

“Universidade Federal Rio Grande do Sul (Brasilia).”

The corrected sentence appears below:

“Federal University of Rio Grande do Sul (Brazil).”

The authors apologize for this error and state that this does not change the scientific conclusions of the article in any way. The original article has been updated.

